# Changes in ecosystem services and an analysis of driving factors for China's Natural Forest Conservation Program

**DOI:** 10.1002/ece3.4925

**Published:** 2019-03-09

**Authors:** Longsheng Huang, Bing Wang, Xiang Niu, Peng Gao, Qingfeng Song

**Affiliations:** ^1^ Research Institute of Forest Ecology, Environment and Protection, Chinese Academy of Forestry, Key Laboratory of Forest Ecology and Environment State Forestry Administration Beijing China; ^2^ Beijing Collaborative Innovation Center for Eco‐Environmental Improvement with Forestry and Fruit Trees Beijing China; ^3^ Shandong Agricultural University, Mountain Tai Forest Ecosystem Research Station of State Forestry Administration Shandong Provincial Key Laboratory of Soil Erosion and Ecological Restoration Tai'an China

**Keywords:** ecological effect, ecosystem services, key state‐owned forest areas, Natural Forest Conservation Program (NFCP), Northeast and Inner Mongolia

## Abstract

China's Natural Forest Conservation Program (NFCP) is aimed at improving the fragile and unstable ecological environment and has become one of the largest ecological restoration programs in the world because of its enormous investment and effects. It is important to work out and strengthen new measures to overcome difficulties to promote more ecosystem services and human well‐being in the NFCP. This study focused on how to evaluate the ecosystem services change brought about by implementing the NFCP. Taking the key state‐owned forest areas in the Northeast and Inner Mongolia as the study area, we provide a basic overview of development and construct an evaluation index system and a distributed calculation method for the NFCP to analyze the implications of the NFCP on ecosystem services combined with multi‐source data coupling. An evaluation index system for NFCP ecosystem services was constructed. The system includes five ecological service functions and 12 evaluation indices. The trade‐off and/or synergistic analysis of ecosystem services were carried out. The regional characteristics and changes in the NFCP ecosystem services were emphasized. Although it has not been implemented for a long time, the NFCP has had a great impact on ecosystem services because it reduces soil and water losses, increases soil fertility, strengthens the forest carbon sink and helped the forest accumulate nutrients and purify the atmosphere. Socioeconomic factors affect the NFCP ecosystem services, such as the implementation area of NFCP, investment amount of NFCP, area ratio of nature reserves, and yield of tree stock volume. Policy drivers of the NFCP, changes in the economic structure and reductions in forest yield are the main factors affecting the change in NFCP ecosystem services. Although the NFCP has positively affected the society, the economy, and the ecological environment, it has also generated some problems, such as the improper management of forest resources, shortage of capital investment, staff transfer, etc. The social and economic problems will be transient with implementation of the NFCP, and the structural changes in forestry and agriculture may eventually benefit the forestry workers and other stakeholders.

## 1 INTRODUCTION

Ecosystem services are constituted by the close association between environmental conditions and human well‐being (Tallis, Goldman, Uhl, & Brosi, 2009). However, the increased social and economic activities of humans have led to degradation of the ecological environment, resulting in many challenges for global sustainable development (Mee, Dublin, & Eberhard, 2008), and nearly 60% of the degradation is unsustainable in ecosystem services (MEA (2005)). Human activities, particularly agricultural expansion and deforestation have covered half of the Earth's land area (Kareiva, Watts, McDonald, & Boucher, 2007; Vitousek, Mooney, Lubchenco, & Melillo, 1997). As forests have functions of soil and water conservation, carbon sequestration, and biodiversity conservation (Foley et al., 2005; Hansen et al., 2013), their degradation will become the most harmful of human activities (Crowther et al., 2015). Under such a challenging background, ecological restoration and protection have been widely recognized and implemented and are based on various forms of ecological restoration projects (Allan et al., 2013; Bullock, Aronson, Newton, Pywell, & Rey‐Benayas, 2011). The China's Natural Forest Conservation Program (NFCP) was launched after China suffered a great flood in 1998 and was a huge project for protecting and restoring the forests. Natural forest resources are effectively protected to prohibit commercial felling of natural forests and forest coverage and ecosystem services have improved (NDPRC, 2009). In addition, the implementation of NFCP has a certain global significance, although it was initially cope with the declining ecological environment in China. However, if implemented reasonably and continuously, it will have an important impact on China and the rest of the world. This study focuses on how to quantitatively evaluate the implications of the ecosystem services.

In recent years, the research of natural forest resources protection projects has emerged in the world. The main research direction can be summed up in four aspects: the practical research on the conservation project of natural forest resources in China, the past experience and future focus of natural forest conservation projects in the world, study on the relationship between conservation of natural forest resources and biodiversity and ecological protection in developing countries (Chen, Cao, & Su, 2017). In the past decades, there has been no interruption in the research of natural forest resources protection program. With the continuous deepening of people's understanding of it, the focus of its research is also changing. In the years after China implemented the natural forest protection project in 1998, due to its large scale, long span and huge investment, it has become the focus of academic research. The research in this period is mainly focused on the policy background, afforestation measures, management methods, and effects of natural forest resources protection projects in China (Lewis, 2005; Lu & Zhang, 2003). As the research goes deep, relevant scholars have studied the protection of natural forest resources for biodiversity conservation and effective land use, and gradually turned from macro to microcosmic (Hannon & McCallum, 2006; Khanina, Bobrovsky, Komarov, & Mikhajlov, 2007). Then, people realize that the important role of this program in carbon fixation has become a hot spot of research (Ryan et al., 2010; Soares‐Filho et al., 2010). Later, the ecosystem service value of this project and the restoration of forest vegetation have become the focus of attention (Santopuoli, 2012; Zhou, Svensson, Yan, Chen, & Li, 2014). Consequently, more people are paying close attention to how to quantitatively assess the impact of the NFCP on ecosystem services, how implementing the NFCP has changed ecosystem services, and the key drivers of the impact (Yu & Han, 2016). But for many reasons, such as lack of unified evaluation index system, complexity of social economy, and lack of reference materials, making such a quantitative assessment very challenging.

National key ecological restoration projects have had an important impact on China's degraded ecosystems, and are the focus of current research. Studies have shown that terrestrial ecosystems in China are important carbon sinks, and that any increase in carbon storage is largely attributed to climate change, ecological restoration projects, and agricultural land management (Fang, Yu, Liu, & Chapin, 2018). For example, ecological restoration projects have contributed to an increase in carbon storage of 74 Tg C/year (Lu et al., 2018). From 1978 to 2015, China has invested more than USD 370 billion across 6.2 million km^2^ (65% of the country's total area) of land through 16 major ecological restoration programs. More than 500 million people have participated in them. Their implementation has resulted in very positive impacts: for example, forests now cover over 22% of China's land area, grasslands have expanded and regenerated, and soil erosion and sedimentation have been significantly reduced. The sediment load of the Yellow River has dropped by 90%, agricultural productivity has grown by 5% annually on average, and millions of farmers have been lifted out of poverty (Bryan et al., 2018). The experiences and achievements gained by many major ecological restoration programs in China help improve future sustainable development projects and policies, as well as to provide examples and lessons for sustainable development in other countries (Sun et al., 2018).

In order to reveal the impact of ecological restoration projects on regional ecosystem services, further strengthening of regional ecological management will be generated based on the Northeast and Inner Mongolia key state‐owned forest region as the study area and the status quo of the NFCP, according to the People's Republic of China Forestry Standard: Forest Ecosystem Service Valuation Norms (LY/T1721‐2008), which was issued by the State Forestry Administration, P.R. China in 2008 (SFAC, 2008). The following research objectives were addressed in this study: (a) analyze the changes in ecosystem services of the NFCP from multivariate data; (b) demonstrate the key drivers for change in NFCP ecosystem services; and (c) discuss the impact of drivers on ecosystem service functions, to clarify the existing problems and challenges, and to provide suggestions for overcoming the difficulties and enhancing their potential. The results will provide a reference for the development, management, and evaluation of similar ecological restoration projects in China and other countries worldwide.

## 2 MATERIALS AND METHODS

### 2.1 Study area

**Figure 1:**
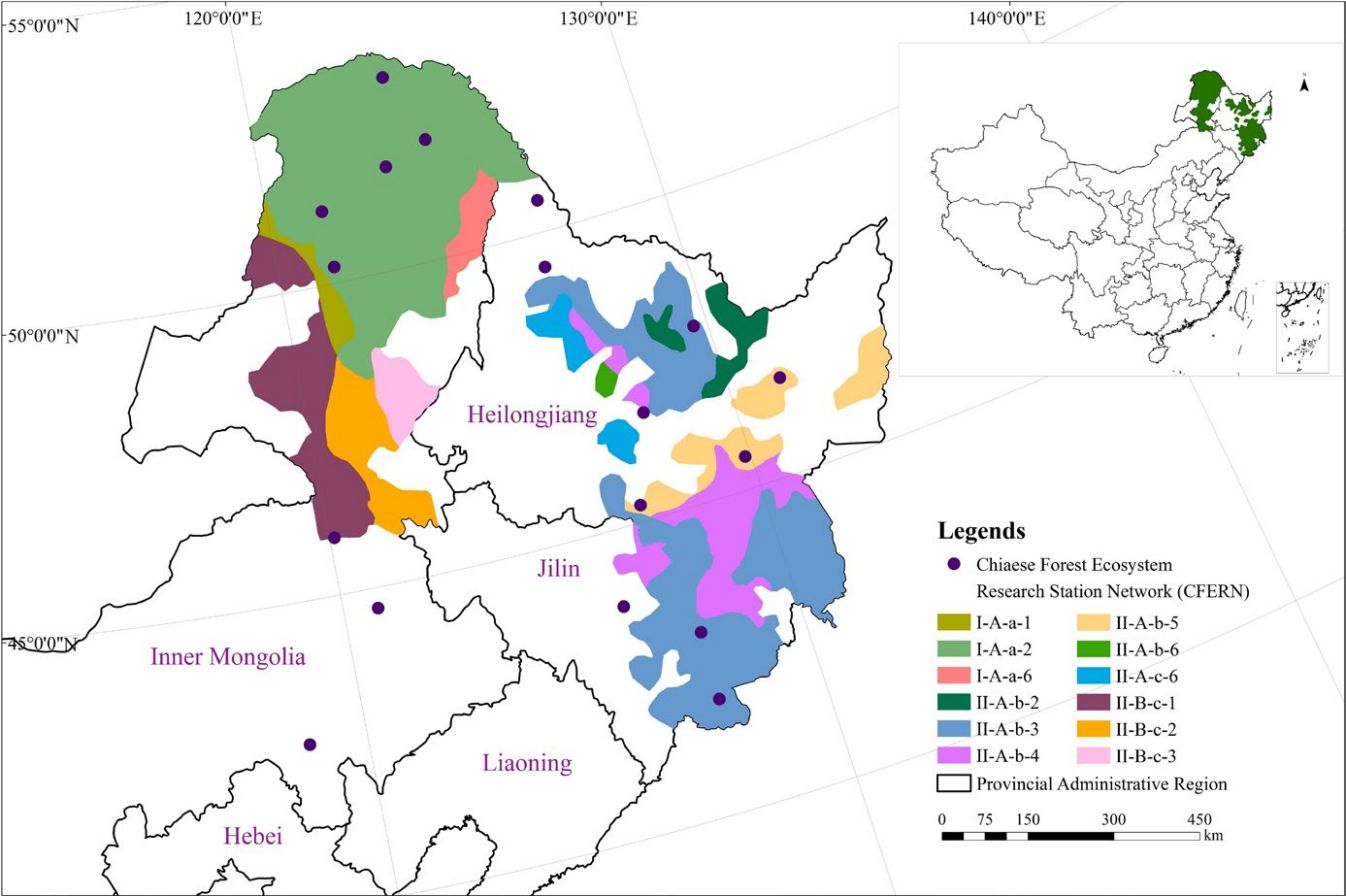
Layout of the forest ecological stations belongs to CFERN of the ecosystem services and the ecological functions monitoring and the assessment area of the NFCP in the key state‐owned forest areas in Northeast and the eastern of Inner Mongolia. CFERN: Chinese Forest Ecosystem Research Station Network. NFCP: Natural Forest Conservation Program. The following the same. I‐A‐a‐1: Micro wind erosion area of deciduous coniferous forest in cold temperature zone; I‐A‐a‐2: Non erosive of deciduous coniferous forest in cold temperature zone; I‐A‐a‐6: Intensity water erosion area of deciduous coniferous forest in cold temperature zone; II‐A‐b‐2: No erosion area in humid temperate coniferous and broad‐leaved mixed forest; II‐A‐b‐3: Micro water erosion area of coniferous and broad‐leaved mixed forest in middle temperate; II‐A‐b‐4: Mild water erosion area in humid temperate coniferous and broad‐leaved mixed forest; II‐A‐b‐5: Moderate water erosion area in coniferous and broad‐leaved mixed forest of moderate temperate zone; II‐A‐B‐6: Intensity of water erosion area in humid temperate coniferous and broad‐leaved mixed forest; II‐A‐c‐6: Moderate humid grassland in moderate intensity water erosion area; II‐B‐c‐1: Micro wind erosion region in mid temperate semi humid grassland; II‐B‐c‐2: No erosion area in mid temperate semi humid grassland; II‐B‐c‐3: Micro water erosion region in mid temperate semi humid grassland. The following the same

The key state‐owned forest areas in the Northeast and Inner Mongolia are mainly distributed in Heilongjiang, Jilin and east of the Inner Mongolia Autonomous Region Province**(**Figure 1), located at 117°06′–135°05′E, 41°25′–53°23′N. The main geomorphic types included mountains, hills, plains, and plateaus, from 30 to 1,600 m. The region is located in the eastern part of the Eurasian continent, most of which belongs to the central temperate zone. Only a few parts of the north belong to the cold temperate zone. The soil types are dominated by dark brown soil, black soil, and brown forest soil. The vegetation types mainly include cold temperate coniferous forest, temperate coniferous and broad‐leaved mixed forest, and plain‐forest grassland (Zhang, Tang, & Lei, 2011).

The NFCP covers the key state‐owned forest areas in Northeast China and Inner Mongolia provinces with a total area of 3,589.97 × 10^4^ha, accounting for 3.74% of the national land area. Since implementation of the NFCP, forest resources have received effective protection and restoration to the NFCP requirements, and commercial logging has stopped; therefore, the forests area and volume have increased from 2,673.42 × ha10^4^ha to 2,788.72 × ha10^4^ha and 18.96 × 10^8^m^3^ to 23.30 × 10^8^m^3^, respectively (Table 1).

**Table 1:** Forest resources of implementation area of NFCP in key state‐owned forest areas in Northeast and Inner Mongolia

Stand age	Year 2000				Year 2015			
	Area (10^4 ^ha)	Proportion (%)	Volume (10^4^ m^3^)	Proportion (%)	Area (10^4^ ha)	Proportion (%)	Volume (10^4^ m^3^)	Proportion (%)
Total	2,673.42	100.00	189,759.64	100.00	2,788.27	100.00	232,973.60	100.00
Young forest	705.97	26.41	28,754.48	15.17	505.48	18.13	22,518.09	9.67
Middle aged forest	1,194.78	44.69	88,361.09	46.61	1520.42	54.53	131,050.14	56.25
Near‐mature forest	323.59	12.10	27,580.74	14.55	432.44	15.51	41,796.20	17.94
Mature forest	357.25	13.36	34,713.79	18.31	263.63	9.45	28,594.15	12.27
Over‐mature forest	91.83	3.44	10,169.54	5.36	66.30	2.38	9,015.02	3.87

### 2.2 Ecological function monitoring and layout of the assessment area

The main indicators of ecological function monitoring and the assessment area according to the differences in natural conditions of the natural forest protection projects in key state‐owned forest areas in Northeast and Inner Mongolia are temperature and water(Zheng, 2008), an index of vegetation regionalization(Zhang, 2007), and soil erosion indices(Li, Zhao, & Yang, 2014). Geometric rectification and vectorization were carried out by defining the projection uniformly in ArcGIS10.0, and then the NFCP implementation scope layer, different temperature and moisture distribution layer regions of China, Chinese vegetation distribution layer, and China soil erosion distribution layer were obtained. Finally, the corresponding layers of Northeast China and Inner Mongolia area were defined, and the ecological function monitoring and evaluation regionalization of the study area was generated (Figure 1).

### 2.3 Forest ecology observation station

Some of the current forest ecosystem stations have regarded NFCP ecosystem services as one of the main monitoring objectives (Wang, Cui, & Yang, 2004). The network layout for the forest ecology station was guided by typical sampling, based on the water and heat distribution and the conditions at the forest site and typical, representative areas were selected to complete the forest ecological network layout (Guo, Wang, & Niu, 2015). Figure 1 shows the forest ecology station responsible for monitoring the ecosystem services of the NFCP in the key state‐owned forest areas in Northeast China and eastern Inner Mongolia. At least 10% of the counties or forestry bureaus in the NFCP area are under long‐term observations to ensure observational accuracy and collect sufficient observational data. There are eight forest industry groups, 41 counties/cities, 100 forestry bureaus, and 18 forest ecology stations (Niu, Wang, & Wei, 2013) in the study area.

### 2.4 Data sources

The data sources for the changes in physical measurements of the NFCP's ecosystem in Northeast China and the Inner Mongolia key state‐owned have three main aspects: (a) a continuous ecological inventory from the study area and the vicinity of the 18 forest ecology stations taking long‐term observation results; (b) a continuous inventory of forest resources in key state‐owned forest areas in Northeast China and Inner Mongolia Province issued by the State Forestry Administration; and (c) public data from the departments of the state (NBSPRC, 2016), the website of the Ministry of Agriculture of China (http://www.agri.gov.cn/), the website of the Ministry of Health of China (http://wsb.moh.gov.cn/), and others. The three types of data sources were coupled and integrated into a series of evaluation formulas (which are detailed in Tables 2 and 3). Finally, the quality and quantity of the NFCP ecosystem services in the study area were evaluated.

**Table 2:** Models of the ecosystem services evaluation of NFCP

Evaluation index	Formula	Parameter descriptions
Water quantity regulation and purification	*G*_WAC_ = 10*A*·(*P*–*E*–*C*)·*F*	*G*_WAC_ is the quantity of water regulated (or water quality purification) for NFCP per year (m^3^/a); *P* is precipitation in open land (mm/a); *E* is evapotranspiration of trees and other plants in the stand (mm/a); *C* is surface runoff (mm/a); and *A* is the area of NFCP (ha); *F* is forest ecological function correction coefficients (*FEFCC*).The following is the same
Decrease of soil loss	*G*_DSL_ = *A*·(*X*_2_−*X*_1_)·*F*	*G*_DSL_ is the quantity of fixed soil per year (t/a); *X*_1_ and *X*_2_ are the modulus of soil erosion of forestlands and non‐forestlands for NFCP (t [hm^−2^ a^−1^]).The following the same
Protection of soil fertility	*G*_PSF_ = *A*·(N + P + K + M)·(*X*_2_−*X*_1_)·*F*	*G*_PSF _is the quantity of fertility maintained for NFCP per year (t/a); N is the concentration of nitrogen in forest soil of NFCP (%); P is the concentration of phosphorus in forest soil of NFCP (%); K is the concentration of potassium in forest soil of NFCP (%); M is the concentration of organic matter in forest soil of NFCP (%)
Carbon fixation	*G*_CAF_ = *A*·(1.63·*R*_c_·*B*_a_ + *F*_sc_)·*F*	*G*_CAF_ is the quantity of carbon fixation for NFCP per year (t/a); *R*_c_ is the carbon content in carbon dioxide, it is 27.2%; *B*_a_ is the net primary productivity of the forest for NFCP (t·(hm^−2^·a^−1^)); *F*_sc_ is the carbon sequestrated in the soil per unit area for NFCP per year(t·(hm^−2^·a^−1^)). The following the same
Oxygen released	*G*_OXR_ = 1.19·*A*·*B*_a_·*F*	*G*_OXR _is the quantity of oxygen released for NFCP per year (t/a).
Nutrients accumulation	*G*_NUA_ = *A*·(N_f_ *+* P_f_ *+* K_f_)·*B*_a_·*F*	*G*_NUA _is the quantity of N, *P*, and *K* accumulated in a stand type of NFCP (t/a); N_f_, P_f_ and K_f_ are the concentrations of N, P, and *K* in dry matter (%)
Negative ion supply	*G*_NIS_ = 5.256.10^15^·*Q*_a_·*A*·*H*·*F*/*L*	*G*_NIS_ is the number of negative ion produced for NFCP per year (N/a); *Q*_a_ is the concentration of anions in the atmosphere in a stand type (ge/cm^3^); *H* is the height of trees for NFCP (m); *L* is the lifetime of a negative ion in the air for NFCP (min)
Pollutant absorption	*G*_POA_ = *A*·*F*(*Q*_SO2 _ *+* *Q*_F_ *+* *Q*_NOx_)/1,000	*G*_POA_ is the quantity of pollutants absorbed for NFCP per year (t/a); *Q*_SO2_ is the quantity of sulfur dioxide absorbed for NFCP per unit area per year (kg·(hm^−2^ a^−1^)); *Q*_F_ is the quantity of fluoride absorbed for NFCP per unit area per year (kg·(hm^−2^ a^−1^)); *Q*_NOx_ is the quantity of nitrogen oxides absorbed for NFCP per unit area per year (kg·[hm^−2^ a^−1^])
TSP absorption	*G*_TSP_ = *Q*_d_ ·*A*·*F*/1,000	*G*_TSP_ is the quantity of TSP absorbed for NFCP per year (t/a); *Q*_d_ is the quantity of dust absorbed for NFCP per unit area per year (kg·[hm^−2^ a^−1^]). The following the same
PM_10_ absorption	*G*_PM10_ = 10·*Q*_PM10_·*A*·n·*F*·LAI	G_PM10_ is the quantity of *PM*_10_ absorbed for NFCP per year (t/a); *Q*_PM10_ is the measured absorption of PM_10_ in the unit area of the stand (kg/a); LAI is leaf area index. The following the same
PM_2.5_ absorption	*G*_PM2.5_ = 10·*Q*_PM2.5_·*A*·n·*F*·LAI	G_PM2.5_ is the quantity of PM_2.5_ absorbed for NFCP per year (t/a); *Q*_PM2.5_ is the measured absorption of PM_2.5_ in the unit area of the stand (kg/a)

Notes

CAF: Carbon fixation, it is the sum of plant carbon fixation and soil carbon fixation; DSL: Decrease of soil loss; NUA: Nutrients accumulation, it is the sum of accumulation of nitrogen, phosphorus, potassium; NIS: Negative ion supply; OXR: Oxygen release; POA: Pollutants absorption, it is the sum of absorption of sulfur dioxide, fluoride and nitrogen oxides; PM_10_: PM_10_ absorption; PM_2.5_: PM_2.5_ absorption; PSF: Protection of soil fertility, it is the sum of soil nitrogen, phosphorus, potassium and organic matter content; TSP: Total suspended particulate absorption; WAC: Water conservation. The following the same.

**Table 3:** Index system of influence on ecosystem services of NFCP based on DPSIR‐mDSS model

Indices		Property	Measurement unit	Source of the data
D_1_	Implementation area of NFCP	S, E_co_, E_nv_	10^4^ha	Data of the continuous investigation of NFCP; Forest resources inventory data
D_2_	Investment amount of NFCP	S, E_co_	10^8^ yuan	Chinese forestry statistical yearbook(2000–2015)
D_3_	Average annual wage of forest workers	S, E_co_	yuan/a	China Forestry Statistical Yearbook(2000–2015)
D_4_	Proportion of industrial and agricultural production	S, E_co_	%	China Statistical Yearbook(2001–2016)
D_5_	Annual average precipitation	E_nv_	mm	China Statistical Yearbook(2001–2016)
D_6_	Annual average temperature	E_nv_	°C	China Statistical Yearbook(2001–2016)
P_1_	Number of forestry workers	S, E_co_	person	China Forestry Statistical Yearbook(2000–2015)
P_2_	Road net density	S, E_co_	m/ha	China Forestry Statistical Yearbook(2000–2015)
P_3_	Yield of tree stock volume	S, E_co_	10^4^m^3^	China Forestry Statistical Yearbook(2000–2015)
P_4_	Area ratio of nature reserves	S, E_co_, E_nv_	%	China Forestry Statistical Yearbook(2000–2015)
P_5_	Natural deviation degree	S, E_co_, E_nv_	%	China Statistical Yearbook(2001–2016)
S_1_	Per unit area of tree stock volume	E_nv_	m^3^/ha	China Forestry Statistical Yearbook(2000–2015)
S_2_	Forest coverage	E_nv_	%	Data of the continuous investigation of NFCP; Forest resources inventory data
S_3_	Forest tending area	E_nv_	ha	China Forestry Statistical Yearbook(2000–2015)
S_4_	Age structure of forest	E_nv_		Data of the continuous investigation of NFCP; Forest resources inventory data
I_1_	Water conservation	E_nv_	10^8^ m^3^	Data of the continuous investigation of NFCP; Forest resources inventory data
I_2_	Soil conservation	E_nv_	10^4^t	Data of the continuous investigation of NFCP; Forest resources inventory data
I_3_	Carbon fixation and oxygen release	E_nv_	10^4^t	Data of the continuous investigation of NFCP; Forest resources inventory data
I_4_	Pollutant Absorption	E_nv_	10^4^kg	Data of the continuous investigation of NFCP; Forest resources inventory data
I_5_	TSP absorption	E_nv_	10^8^kg	Data of the continuous investigation of NFCP; Forest resources inventory data
I_6_	Biodiversity conservation	E_nv_		China Forestry Statistical Yearbook(2000–2015)
I_7_	Ecosystem services value	S, E_co_, E_nv_	10^8^yuan	Data of the continuous investigation of NFCP; Forest resources inventory data

Note

E_co_: Economic; E_nv_: Environment; S; Society.

### 2.5 Evaluation index system of ecosystem services for the NFCP

**Figure 2:**
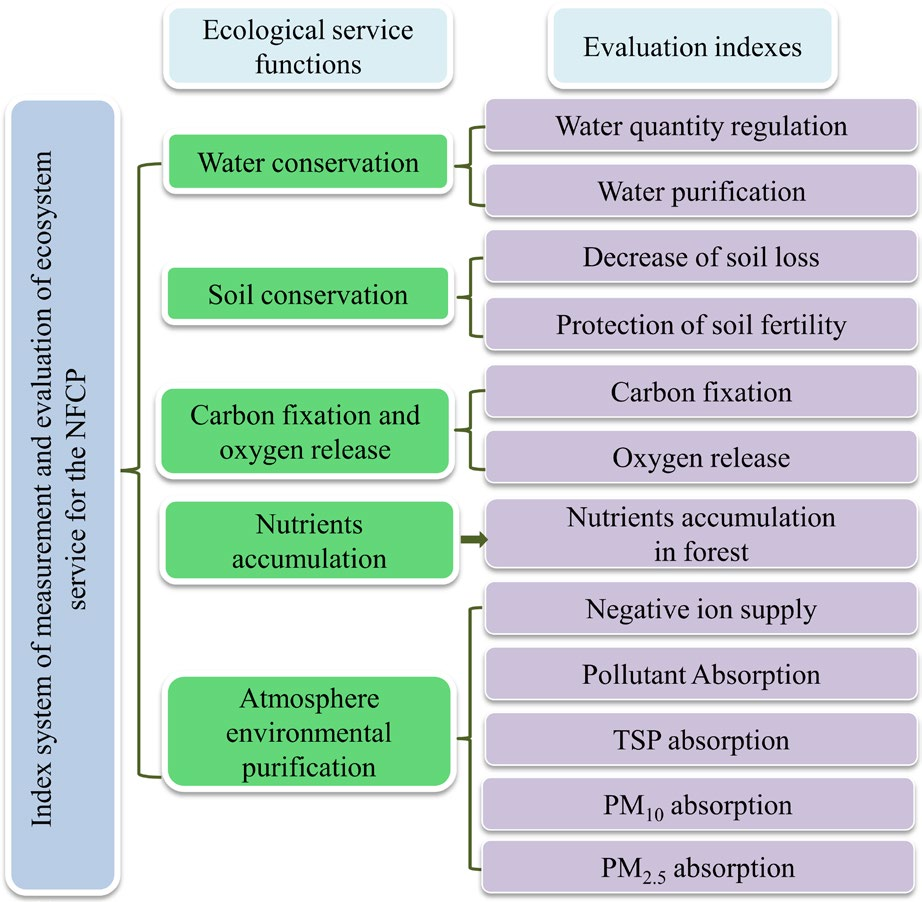
Evaluation index systems of the ecosystem services of NFCP

The evaluation index system of ecosystem services for the NFCP was determined based on the evaluation standard of forest ecosystem services (LY/T1721‐2008) and long‐term observations of the forest ecosystem (Wang et al., 2003). The index system included five ecological service functions and 12 evaluation indices (Figure 2). The five ecological service functions are water conservation (water quantity regulation and water purification), soil conservation (decrease soil loss and protection of soil fertility), carbon fixation and oxygen release, nutrients accumulation in the forest, atmospheric environmental purification (negative ion supply, pollutant absorption, total suspended particulate(TSP) absorption, PM_10 _absorption, and PM_2.5_ absorption; Wang et al., 2011). Table 2 shows the model, formulas, and definition of the 12 evaluation indicators for NFCP ecosystem services (Wang et al., 2003).

### 2.6 Trade‐off and/or synergies analysis of ecosystem services for the NFCP

Zoning statistics of ecosystem services were carried out based on the unit of the forestry bureau in the NFCP area. The values were standardized, and the changes over the whole research period (2000–2015) were calculated. Finally, the changes were imported into SPSS for bivariate correlation analysis, and correlation coefficients between water conservation, soil conservation, carbon sequestration and oxygen release, tree nutrient accumulation, and air purification were obtained (Table 6).

### 2.7 Analysis of factors influencing NFCP ecosystem services based on the DPSIR‐mDSS model

Driving force‐Pressure‐State‐Impact‐Response (DPSIR) is a conceptual framework used to reveal the causal relationship between the state of the environment and human activities, and has been widely used in the study of resource management, population, and sustainable development (Benini, Bandini, Marazza, & Contin, 2010; Burkhard & Müller, 2008; Fassio, Giupponi, Hiederer, & Simota, 2005; Malekmohammadi & Jahanishakib, 2017). Because of the different evaluation objects and emphasis, the DPSIR conceptual framework often has great differences in the evaluation indexes and methods of the ecological environment response assessment, but its core idea is that natural forces and human activities (driving forces) constitute a huge “pressure” on the ecosystem, shifting the ecological environment into a deteriorated “state”. This state produces an unsustainable "impact," which human beings must take certain measures (responses) to reduce and thus achieve sustainable development (EEA, 2005; OECD, 1991). In a project of sustainable use of water resources at the river basin scale, the European Union tried to combine the multi‐project integrated decision support system (mDSS) with the DPSIR conceptual framework, and achieved good results. The project MULINO, funded by the European Commission, has released a prototype of Decision Support System software (mDSS) for sustainable management of water resources at watershed scale. The software integrates socioeconomic, environmental, geospatial information, and multi‐criteria analysis for modeling. Through the DPSIR framework originally proposed by the European Environment Agency, challenging interdisciplinary issues have been innovatively addressed. Therefore, the combination of DPSIR and mDSS model provides users with integrated visualization of complex interaction problems. The mDSS model provides quantitative indicators for decision‐making by applying value function, weight and decision rules of end‐user selection. The model is used for sensitivity analysis and comparison of alternative weights to explore and find trade‐offs among multiple stakeholders, thus providing effective decision support for watershed water resources management (Giupponi, Mysiak, Fassio, & Cogan, 2004; La Jeunesse, Rounsevell, & Vanclooster, 2003).

The DPSIR conceptual framework was used as the evaluation method, and the results of this evaluation were then quantified using the mDSS method. Then, the combined DPSIR‐mDSS model has been widely used in sustainable development assessment, strategic environmental impact assessment, urban and rural development assessment, climate change adaptation strategy, and ecological agriculture system assessment. In light of this, this paper attempts to construct a DPSIR conceptual framework for the evaluation of the environmental effects of the NFCP, and combined with the mDSS model, analyzes the factors influencing ecosystem services in the key state‐owned forest areas of Northeast and Inner Mongolia in 2000 and 2015.

**Figure 3:**
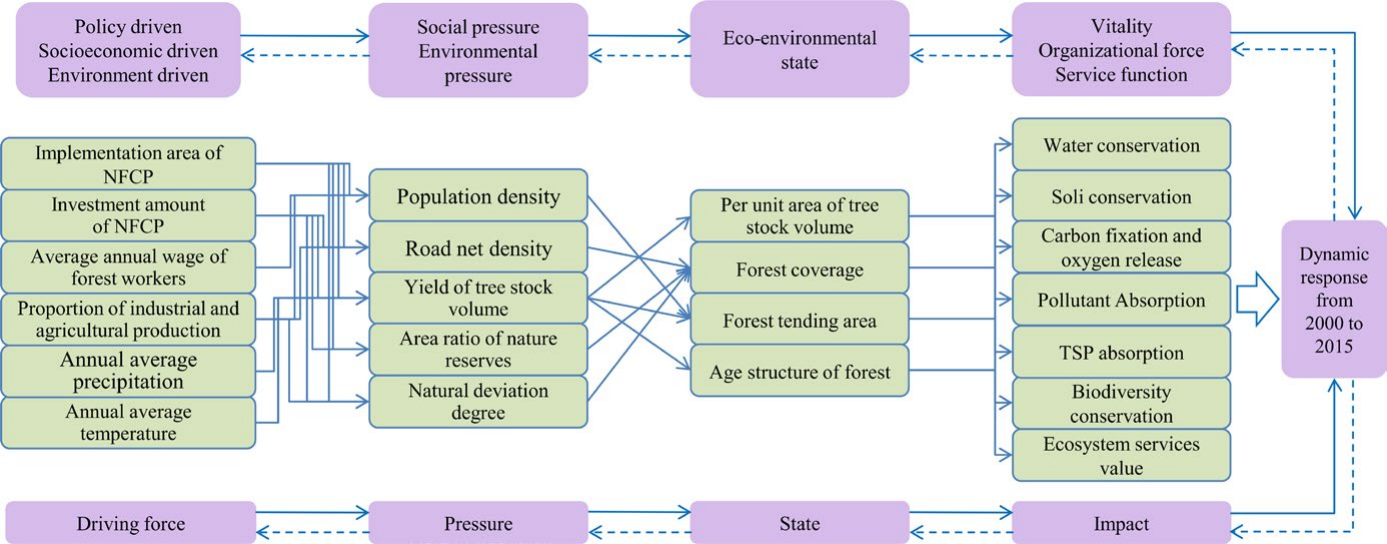
Evaluation index system of ecosystem services based on the conceptual framework of DPSIR for the NFCP

The NFCP in key state‐owned forests is the most important policy for protection of forest resources at a regional level. In the northeastern provinces of Jilin, Liaoning, Heilongjiang, and Inner Mongolia, it has played a positive role in local environmental restoration. The continuing development of the social economy (average annual wage of forest workers, amount invested in NFCP, etc.) and associated changes of people's thinking about and behavior toward the environment in this region affect the relationship between people and the ecosystem. Climate factors (annual average precipitation, effective accumulated temperature >10°C, etc.) also have an impact on the local environment, and are in turn driven by climate change. Together, these driving forces exert positive or negative pressures on the regional ecological environment (population density, road net density, yield of tree stock volume, area ratio of nature reserves, proportion of industrial and agricultural production, etc.), thus affecting the state of the local ecosystem (per unit area of tree stock volume, forest coverage, etc.). This in turn affects human and ecosystem health and human well‐being. Policymakers and stakeholders should then design appropriate policies (e.g., NFCP) and economic measures to offset or control these adverse impacts, based on the impact of human or climate on the region. This process is described, quantified, and monitored by some evaluation indicators, which constitutes the conceptual framework of the Driving Force Pressure State Impact Response in the process of vegetation restoration in the key state‐owned forest areas of Northeast and Inner Mongolia (Figure 3).

The specific evaluation methods (Giupponi et al., 2004) were divided into the following four stages:

1. Conceptualization phase. We selected 22 indicators (six driving indicators, five pressure indicators, four state indicators, and seven impact indicators) **(**Table 3 **). **The indices were screened, and causal indicators were constructed according to the DPSIR conceptual framework (Figure 3).
2. Design phase. The evaluation index was digitally standardized by the mDSS model. The indices were divided into an ascending distribution function (Equation (1)), descending distribution function (Equation (2)), and an interval distribution function (Equation (3)), and digital regulation was adopted to obtain the subordinate degree of each index.

(1) $$u_{\mathrm{ij}}=\frac{x_{\mathrm{ij}}-x_{\min}}{x_{\max}-x_{\min}}$$

(2) $$u_{\mathrm{ij}}=\frac{x_{\max}-x_{\mathrm{ij}}}{x_{\max}-x_{\min}}$$

(3) $$u_{\mathrm{ij}}=\left\{\begin{array}{cc} 1 & b_{2}\geq x_{\mathrm{ij}}\geq b_{1}\\ \frac{x_{\mathrm{ij}}-a_{1}}{b_{1}-a_{1}} & a_{1}<x_{\mathrm{ij}}<b_{1}\\ \frac{x_{\mathrm{ij}}-a_{2}}{b_{2}-a_{2}} & a_{2}>x_{\mathrm{ij}}>b_{2}\\ 0 & x_{\mathrm{ij}}\leq a_{i}orx_{\mathrm{ij}}\geq a_{2} \end{array}\right.$$

where *x_ij_* corresponds to option *(i)* and criterion *(j)*, *x*_min _and *x*_max_ are the lowest and highest scores of the *j*th criterion, *a*_1 _and *a*_2 _represent the lower and upper bounds of the critical value of the index, respectively, and *b*_1 _and *b*_2 _represent the upper and lower bounds of the optimal values of the point, respectively.

The implementation area of NFCP, proportion of industrial and agricultural production, number of forestry workers, road density, area of nature reserves, forest coverage, and age structure of forest were selected as interval indices. We used the interval distribution function to calculate group membership (Equation (3)). The implementation area of NFCP takes the national forest resources area and the local forest resources area as the interval range. The proportion of industrial and agricultural production is in the range of national industrial and agricultural production and local industrial and agricultural production. The number of forestry workers takes the national number of forestry workers and the local number of forestry workers as the interval range. The road network density takes the national road network density and the local road network density as the interval range, and the area ratio of the naturreserves takes the area ratio of the National Nature Reserves and the area ratio of the local nature reserves as the interval range. Forest coverage takes 1 as the interval point. The proportion of age structure of forest resources in China and that of local forest resources were taken as the interval range of age structure. The investment amount of NFCP, average annual wage of forest workers, annual average precipitation, annual average temperature, per unit area of tree volume, forest tending area, water conservation, soil conservation, carbon sequestration and oxygen release, pollutant absorption, dust retention, biodiversity protection, and ecosystem service value were selected as positive indicators. The ascending type distribution function calculates its membership degree (Equation (1)). Natural deviation degree and per unit area of tree stock volume were selected as negative indices, and the membership degree (Equation (2)) was calculated by the reduced distribution function. The calculation results of membership degree and weights of indices of ecological restoration environmental effects for NFCP were shown in Table 4.

- Choice phase. We formulated the decision criteria for the evaluation by giving weight to the evaluation index. Objective weights were computed by entropy methods (Equations (4), (5), (6), (7), (8)). Then, the model is adjusted by the PWC (Pairwise comparison method), and the final evaluation criteria are obtained by inputting the model with the expert knowledge base.

**Table 4:** Membership degree and weights of indices of ecological restoration environmental effects of NFCP

Indices	Membership degree																Weights
	2000	2001	2002	2003	2004	2005	2006	2007	2008	2009	2010	2011	2012	2013	2014	2015	
D_1_	0.56	0.62	0.64	0.67	0.71	0.75	0.77	0.81	0.86	0.90	0.93	0.93	0.94	0.94	0.94	0.94	0.133
D_2_	0.33	0.36	0.39	0.41	0.45	0.51	0.55	0.60	0.66	0.71	0.77	0.82	0.85	0.90	0.91	0.91	0.116
D_3_	0.30	0.32	0.32	0.35	0.38	0.42	0.44	0.47	0.52	0.58	0.65	0.77	0.80	0.89	0.92	0.92	0.076
D_4_	0.41	0.42	0.45	0.48	0.51	0.53	0.55	0.58	0.62	0.68	0.75	0.83	0.85	0.86	0.87	0.87	0.085
D_5_	0.44	0.50	0.67	0.46	0.58	0.79	0.48	0.56	0.47	0.59	0.70	0.62	0.55	0.72	0.58	0.60	0.017
D_6_	0.58	0.59	0.65	0.70	0.82	0.68	0.57	0.88	0.68	0.62	0.73	0.66	0.78	0.69	0.60	0.71	0.022
P_1_	0.94	0.94	0.94	0.90	0.87	0.87	0.82	0.77	0.75	0.72	0.70	0.68	0.64	0.61	0.61	0.61	0.105
P_2_	0.45	0.46	0.49	0.50	0.50	0.55	0.60	0.61	0.65	0.68	0.70	0.75	0.72	0.70	0.73	0.71	0.081
P_3_	0.91	0.92	0.90	0.89	0.92	0.86	0.80	0.79	0.77	0.68	0.64	0.60	0.52	0.40	0.35	0.31	0.041
P_4_	0.40	0.40	0.42	0.45	0.52	0.54	0.55	0.59	0.60	0.65	0.69	0.73	0.78	0.78	0.80	0.81	0.018
P_5_	0.46	0.47	0.47	0.51	0.55	0.58	0.62	0.68	0.73	0.77	0.82	0.85	0.88	0.86	0.87	0.85	0.092
S_1_	0.42	0.44	0.47	0.47	0.49	0.52	0.53	0.55	0.59	0.62	0.65	0.67	0.67	0.71	0.72	0.74	0.011
S_2_	0.51	0.50	0.55	0.58	0.57	0.60	0.63	0.65	0.67	0.68	0.70	0.75	0.82	0.84	0.87	0.86	0.083
S_3_	0.33	0.34	0.37	0.40	0.45	0.48	0.50	0.58	0.61	0.65	0.73	0.80	0.82	0.85	0.83	0.87	0.012
S_4_	0.55	0.56	0.58	0.62	0.63	0.60	0.64	0.65	0.69	0.70	0.71	0.75	0.80	0.81	0.83	0.82	0.011
I_1_	0.52	0.56	0.56	0.60	0.61	0.61	0.65	0.65	0.68	0.69	0.72	0.72	0.71	0.74	0.74	0.73	0.010
I_2_	0.60	0.60	0.62	0.62	0.62	0.62	0.65	0.66	0.68	0.68	0.74	0.78	0.80	0.80	0.78	0.76	0.013
I_3_	0.55	0.55	0.60	0.61	0.65	0.65	0.68	0.69	0.69	0.74	0.76	0.82	0.85	0.81	0.80	0.83	0.015
I_4_	0.45	0.48	0.53	0.58	0.61	0.62	0.62	0.67	0.70	0.78	0.82	0.80	0.80	0.78	0.82	0.79	0.009
I_5_	0.33	0.37	0.40	0.42	0.43	0.48	0.52	0.55	0.65	0.74	0.77	0.75	0.78	0.74	0.78	0.76	0.013
I_6_	0.57	0.57	0.58	0.60	0.60	0.64	0.64	0.65	0.69	0.70	0.75	0.84	0.88	0.91	0.90	0.93	0.017
I_7_	0.51	0.52	0.55	0.62	0.64	0.66	0.69	0.70	0.73	0.77	0.81	0.85	0.87	0.89	0.90	0.90	0.020

The objective weight of the entropy method is as follows:

(4) $$w_{j}=g_{i}/\sum_{i=1}^{k}g_{i}$$

(5) $$g_{i}=1-e_{j}$$

(6) $$e_{j}=-k\sum_{i=1}^{m}\left(R_{\mathrm{ij}}\cdot \ln R_{\mathrm{ij}}\right)$$

(7) $$R_{\mathrm{ij}}=u_{\mathrm{ij}}/\sum_{i=1}^{m}u_{\mathrm{ij}}$$

(8) $$Q=\sum_{i=1}^{k}\left(w_{i}\cdot u_{i}\right)$$

where *W_j_* is the weight of the *j* index, *k* is the index number, *g_j_* is the *j* index difference coefficient, *e_j_* is the *j* index entropy, *R_ij_* is the proportion of the *j* index, *u_ij_* is subordinate degree, *m* is different evaluation years, *Q* is the comprehensive value of the eco‐environmental effect, *u_i_* is the subordinate value of the *i* evaluation index, and *w_i_* is the weight of the *i* evaluation index.

- Sensitivity analysis. A sensitivity analysis of the evaluation indices was carried out to judge the most sensitive factors affecting the NFCP ecosystem services in key state‐owned forest areas in Northeast and Inner Mongolia, China (Equation (9)).

(9) $$\frac{Q_{j-}Q_{i}}{q_{\mathrm{jk}}-q_{\mathrm{ik}}}\leq w_{k}$$

where *Q_j_* and *Q_i_* represent the composite values of the *j* and *i* schemes, *q_jk_* and *q_ik_* represent the composite values of the index *k* in the *j* and *i* schemes, and *w_k_* is the weight of index *k*.

## 3 RESULTS

### 3.1 Evaluation of ecosystem services during implementation of the NFCP

The evaluation of ecosystem services during implementation of the NFCP in the key state‐owned forest areas in Northeast China and Inner Mongolia is shown in Table 5. The differences in the ecosystem services between 2000 and 2015 were mainly manifested in the following aspects.

**Table 5:** Annual ecosystem services of different ecological function monitoring and assessment areas of the implementation period for NFCP in key state‐owned forest areas in Northeast and Inner Mongolia

Function area	Water conservation			Soil conservation						Carbon fixation and oxygen release						Nutrients accumulation		
	WAC (10^8 ^m^3^)			DSL (10^4^ t)			PSF (10^4^ t)			CAF (10^4 ^t)			OXR (10^4^ t)			NUA (10^4^ t)		
	2000	2015	Increment	2000	2015	Increment	2000	2015	Increment	2000	2015	Increment	2000	2015	Increment	2000	2015	Increment
I‐A‐a‐1	8.09	12.35	4.26	2,139.62	2,479.49	339.87	111.7	142.08	30.38	108.13	144.62	36.49	269.99	349.69	79.7	6.46	9.43	2.97
I‐A‐a‐2	178.33	254.56	76.23	46,966.75	55,518.28	8,551.53	2,849.63	3,766.06	916.43	2,541.09	3,690.42	1,149.33	6,509.35	9,402.67	2,893.32	139.41	224.51	85.1
I‐A‐a‐6	7.18	9.39	2.21	2066.7	2,268.59	201.89	111.18	128.63	17.45	85.23	99.95	14.72	213.23	241.94	28.71	5.51	6.42	0.91
II‐A‐b‐2	9.68	12.06	2.38	1619.07	2050.77	431.7	104.96	129.5	24.54	89.01	94	4.99	233.16	245.2	12.04	5.54	6.54	1
II‐A‐b‐3	185.8	220.07	34.27	33,237.05	41,621.5	8,384.45	2,150.21	2,572.67	422.46	1696.87	1826.86	129.99	4,439.35	4,703.72	264.37	84.99	101.08	16.09
II‐A‐b‐4	48.06	55.97	7.91	8,263.18	10,352.12	2088.94	539.53	628.74	89.21	410.22	460.21	49.99	1,072.36	1,189.81	117.45	21.15	27.07	5.92
II‐A‐b‐5	27.91	34.73	6.82	4,701.44	5,957.39	1,255.95	308.51	367.28	58.77	246.16	256.73	10.57	644.12	669.86	25.74	15.96	17.33	1.37
II‐A‐b‐6	3.6	4.63	1.03	696.65	840.03	143.38	45.74	47.17	1.43	32.76	33.33	0.57	85.51	87.2	1.69	1.73	2.18	0.45
II‐A‐c‐6	12.26	13.53	1.27	1931.28	2,314.08	382.8	122.72	147.52	24.8	106.75	90.96	−15.79	279.66	236.65	−43.01	7.62	6.37	−1.25
II‐B‐c‐1	14.73	24.38	9.65	3,878.24	5,047.5	1,169.26	192.22	267.72	75.5	167.89	248.13	80.24	416.71	594.66	177.95	10.03	15.79	5.76
II‐B‐c‐2	18.08	26.35	8.27	4,899.09	5,382.44	483.35	264.34	323.46	59.12	254.92	334.64	79.72	638.87	817.26	178.39	15.6	22.68	7.08
II‐B‐c‐3	7.92	11.19	3.27	2,242.18	2,578.21	336.03	113.16	140.85	27.69	91.02	123.23	32.21	226.63	298.47	71.84	5.63	8.06	2.43
**Total**	**521.64**	**679.21**	**157.57**	**112,641.25**	**136,410.40**	**23,769.15**	**6,913.9**	**8,661.68**	**1747.78**	**5,830.05**	**7,403.08**	**1573.03**	**15,028.94**	**18,837.13**	**3,808.19**	**319.63**	**447.46**	**127.83**

Function area	Atmosphere environmental purification														
	NIS (10^25^ N)			POA (10^4^ kg)			TSP (10^8^ kg)			PM_10 _(10^4^ kg)			PM_2.5 _(10^4^kg)		
	2000	2015	Increment	2000	2015	Increment	2000	2015	Increment	2000	2015	Increment	2000	2015	Increment
I‐A‐a‐1	0.36	0.56	0.2	8,834.8	9,074.75	239.95	139.69	172.33	32.64	168.86	222.34	53.48	41.24	54.84	13.6
I‐A‐a‐2	11.04	14.88	3.84	181,615.41	193,358.25	11,742.84	2,585.77	3,663.12	1,077.35	5,020.81	5,894.63	873.82	1,281.28	1,447.21	165.93
I‐A‐a‐6	0.27	0.44	0.17	5,788.59	6,098.89	310.3	107.03	131.25	24.22	55.2	87.1	31.9	49.43	52.04	2.61
II‐A‐b‐2	0.41	0.54	0.13	4,908.99	6,315.45	1,406.46	106.36	113.04	6.68	100.61	238.98	138.37	61.78	54.86	−6.92
II‐A‐b‐3	9.66	10.48	0.82	93,843.23	109,415.33	15,572.1	2,352.7	2,477.9	125.2	2,449.15	3,650.11	1,200.96	769.28	931.23	161.95
II‐A‐b‐4	2.35	2.57	0.22	23,797.4	27,918.41	4,121.01	578.5	591.97	13.47	659.18	948.86	289.68	212.24	241.31	29.07
II‐A‐b‐5	1.21	1.49	0.28	14,278.42	18,638.97	4,360.55	292.83	323.32	30.49	329.31	708.67	379.36	144.13	163.97	19.84
II‐A‐b‐6	0.19	0.18	−0.01	2,615.69	2,474.57	−141.12	40.27	42.22	1.95	69.89	85.46	15.57	16.99	19.48	2.49
II‐A‐c‐6	0.51	0.59	0.08	5,680.79	7,866.58	2,185.79	117.11	126.26	9.15	122.59	307.08	184.49	31.19	68.81	37.62
II‐B‐c‐1	0.65	1.05	0.4	14,509.72	16,706.23	2,196.51	233.4	312.05	78.65	227.25	333.86	106.61	59.56	90.41	30.85
II‐B‐c‐2	0.83	1.19	0.36	19,274.74	19,788.47	513.73	312.29	362.91	50.62	360.93	495.08	134.15	112.23	153.21	40.98
II‐B‐c‐3	0.3	0.51	0.21	7,051.28	7,508.63	457.35	121.97	144.87	22.9	82.74	127.32	44.58	40.75	54.06	13.31
Total	27.78	34.48	6.70	382,199.06	425,164.53	42,965.47	6,987.92	8,461.24	1,473.32	9,646.52	13,099.49	3,452.97	2,820.1	3,331.43	511.33

Soil and water losses were reduced. As shown in Table 5, after implementing the NFCP, the quantity of water conservation (WAC) in the study area increased by 157.57 × 10^8^m^3^/a, which was equivalent to 40.09% of the maximum capacity of the Three Gorges reservoir (393 × 10^8^ m^3^) and a 30.21% increase compared with prior to the NFCP. The decrease in soil loss (DSL) increased by 2.38 t/a, equivalent to 44.65% of the total amount of soil erosion (5.34 × 10^8^ t, Bulletin of Soil and Water Conservation in China, 2014; MWRPRC, 2014) of China's 11 major rivers in 2014 and 21.14% higher than before the NFCP.

Increased soil fertility. As shown in Table 5, protection of soil fertility (*PSF*) in the study area increased by 1,747.78 × 10^4^t/a, equivalent to 29.02% of China's fertilizer application rate (6,022.6 × 10^4^t, China Statistical Yearbook, 2016) (NBSPRC, 2016) in 2015, and 25.48% higher than before the NFCP.

Enhanced forest carbon sequestration and accumulated nutrients. As shown in Table 5, after implementing the NFCP, the quantity of carbon sequestration (*CAS*) in the study area increased by 1,573.03 × 10^4^ t/a, equivalent to 0.52% of the total carbon released by China's standard coal consumption (43 × 10^8^ t, China Statistical Yearbook, 2016; NBSPRC, 2016) in 2015, and 26.98% higher than before the NFCP. Nutrients accumulation (NUA) increased by 127.83 × 10^4^t/a, which was 39.99% higher than before the NFCP.

Purify the atmospheric environment. As shown in Table 5, after implementing the NFCP, the quantity of pollutants absorbed (*POA*) in the study area increased by 4.30 × 10^8^kg/a, equivalent to 9.92% of total SO_2_ and NO_x_ emissions (433.56×10^4^kg, China Statistical Yearbook, 2016; NBSPRC, 2016) in China, Inner Mongolia, Heilongjiang, and Jilin Provinces in 2015, and 11.25% higher than that before the NFCP. TSP absorption (TSP) increased by 1,473.32 × 10^8^kg/a, which was equivalent to 9.58 times of the amount of smoke (dust) emissions (1,538.01 × 10^4^t, China Statistical Yearbook, 2016; NBSPRC, 2016) from China in 2015 and 21.08% higher than before the NFCP.

### 3.2 Trade‐off and/or synergies of ecosystem services for the NFCP

Correlation analysis showed that there were strong trade‐offs between water conservation and soil conservation, water conservation and carbon fixation and oxygen release, water conservation and nutrients accumulation, soil conservation and carbon fixation and oxygen release, soil conservation and nutrients accumulation (the highest correlation coefficient was 0.912). However, there is a weak balance between atmosphere environmental purification and the four functions of water conservation, soil conservation, carbon fixation and oxygen release, and nutrients accumulation (the maximum absolute value of correlation coefficient is only 0.226) (Table 6). Specifically, water conservation and soil conservation, water conservation and carbon fixation and oxygen release, water conservation and nutrients accumulation are positively correlated, showing a mutually beneficial synergistic relationship (correlation coefficients were 0.873, 0.682, and 0.578, respectively). Water is one of the key elements in structuring ecosystems and controlling the flow of ecological processes and functions (Fu, Wang, Su, & Forsius, 2013), stable water supply is the basis for maintaining many ecosystem services (Maes et al., 2012). There were positive correlations between soil conservation and carbon fixation and oxygen release, and between soil conservation and nutrients accumulation (correlation coefficients were 0.842 and 0.912, respectively), which showed that stable soil conservation provided a guarantee for carbon fixation of ecosystem and nutrients accumulation. Atmosphere environmental purification is negatively correlated with water conservation, soil conservation, carbon fixation and oxygen release, and nutrients accumulation, which is a trade‐off relationship between growth and decline (correlation coefficients were 0.105, 0.145, 0.094, and 0.226, respectively).

**Table 6:** The relationship among ecosystem services of NFCP

Ecosystem Services	Water conservation	Soil conservation	Carbon fixation and oxygen release	Nutrients accumulation	Atmosphere environmental purification
Water conservation	1.000				
Soil conservation	0.873^a^	1.000			
Carbon fixation and oxygen release	0.682^a^	0.842^a^	1.000		
Nutrients accumulation	0.578^a^	0.912^a^	−0.013	1.000	
Atmosphere environmental purification	−0.105	−0.145	−0.094	−0.226	1.000

a Indicates significant correlation at 0.01 level (bilateral).

### 3.3 Key factors affecting the change in ecosystem services in the NFCP

**Figure 4:**
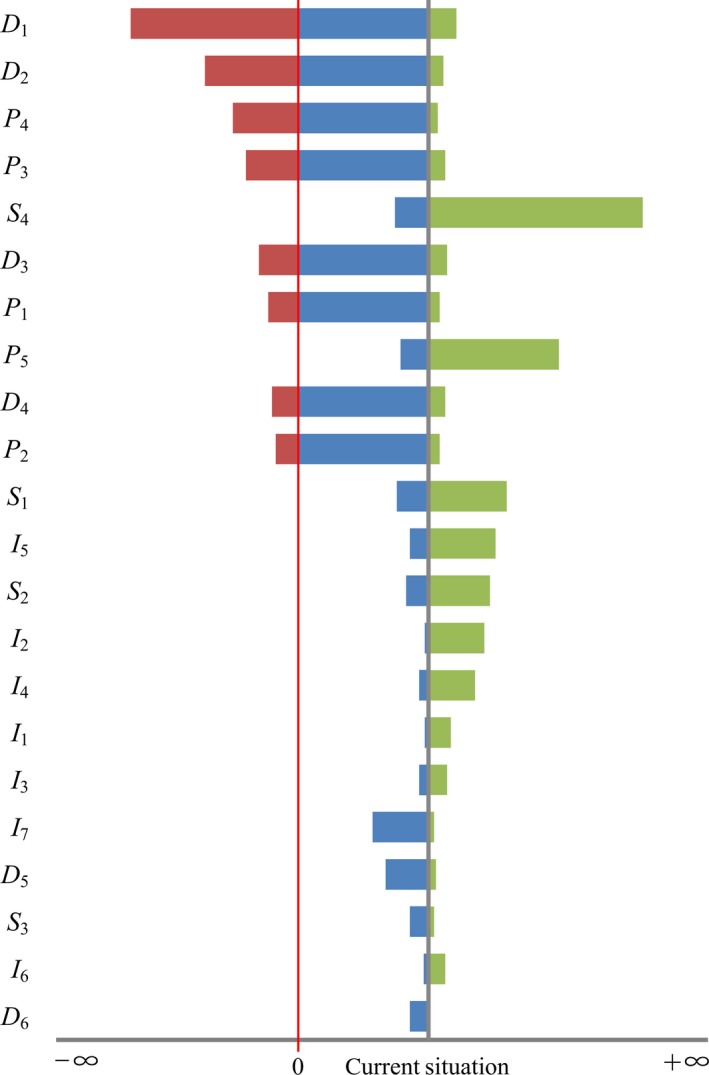
Results of sensitive analysis for the NFCP

Some studies have shown that the socioeconomic (policy, population, economic development) and ecological environments (climate, soil, vegetation) were the two major factors affecting ecosystem services during ecological restoration. This is somewhat similar to the results of this study (Gao, Niu, Wang, & Zheng, 2015; Liu, Li, Ouyang, Tam, & Chen, 2008). A sensitivity analysis (Figure 4) showed that the lateral axis represents the difference between the composite values, and the current situation represents the composite value difference in the contrast scheme. The more comprehensive value difference was less than zero, indicating that the stronger the ability of this factor to change the comprehensive value, the higher the sensitivity. The weights of one factor were changed sequentially, and the weights of other factors remained unchanged. The sensitivity of different factors was obtained by ranking the magnitude of the composite values from top to bottom. The most sensitive factors influencing the ecosystem services of the NFCP were the implementation area of NFCP (*D*_1_), investment amount of NFCP (*D*_2_), area ratio of nature reserves (*P*_4_), yield of tree stock volume (*P*_3_), average annual wage of forest workers (*D*_3_), number of forestry workers (*P*_1_), proportion of industrial and agricultural production (*D*_4_), and road net density (*P*_2_). The factors *D*_1_, *D*_2_, *P*_3_, and *P*_4 _are directly related to the implementation effects of the NFCP and are driving forces of the policy. The factors *D*_3_, *D*_4_, *P*_1_, and *P*_2 _were closely related to construction of forested areas and income of workers, which are representative of social and economic development. The improvement in workers’ incomes gives the forest people higher requirements for the ecological environment, while construction of the forest roads is conducive to the development of forest tourism and other industries, and further enhances the economy of the area (Xie, Zhang, Zhen, & Zhang, 2017; Yin, Yin, & Li, 2010).

Therefore, the socioeconomic factors that most affected NFCP ecosystem services were expanding the proportion of project investment, increasing the area of the nature reserve and reducing forest yield, which directly improved the effectiveness of the NFCP implementation. In contrast, factors such as annual average precipitation, effective accumulated temperature >10°C, the forest coverage and other ecological environmental attributes played a secondary role.

## 4 DISCUSSION

### 4.1 Comments on the DPSIR‐mDSS model

The DPSIR‐mDSS model uses the DPSIR conceptual model to construct the ecosystem service evaluation index system for ecological restoration to explore the causes of ecosystem service status (state), direct factors (pressure), indirect factors (driving force), and ecosystem service status (state) caused by the changes in ecosystem service function (impact), and the relevant measures for the adverse effects (response; Giupponi et al., 2004). In this process, the complex relationship between human behavior and the ecological system becomes a simple cause and effect relationship, making the evaluation process clearer and easier to understand. Because it is a causal relationship, a quantitative index for the interaction between them is possible (Durairaj, Sudharsun, & Swamynathan, 2013). Notably, ecological restoration needs to go through the process within a certain time interval (Celentano & Rousseau, 2016; Wortley, Hero, & Howes, 2013). The comprehensive evaluation results of the DPSIR‐mDSS model better reflect fluctuations in the process of ecological restoration.

### 4.2 Problems and recommendations for the NFCP

The sensitivity analysis based on the model showed that policy and capital input for the NFCP played a pivotal role in ecosystem service changes. The NFCP has had positive impacts on the social, economic, and ecological environments but also faces some problems (Ren et al., 2015; Xu, Yin, Li, & Liu, 2006). The accuracy of ecosystem services valuation is closely related to ecosystem services monitoring, ecosystem services management can provide support for relevant policies, and wages and population density vary with staff turnover. As a factor that influences the change in NFCP ecosystem services, forest yield has a direct relationship with forest resource management and capital investment. Therefore, this study combined factors that impacted ecosystem services change for the NFCP to reveal the problems with the NFCP from the following aspects and puts forward some suggestions.

#### 4.2.1 Special monitoring of ecosystem services

Currently, there are obvious deficiencies in the basic data for the evaluation of the NFCP ecosystem services (Ke, Chen, Robson, Wen, & Tian, 2015; Yin et al., 2010). First, the forest resources data of the basic implementation units need to be determined in the NFCP area. In terms of current technical level and database, it is feasible and necessary to make use of the spatial analysis technology to analyze the shared high resolution remote sensing image data, and determine the NFCP area and the implementation of unit space boundary (Lü et al., 2015; Viña, McConnell, Yang, Xu, & Liu, 2016). Second, no NFCP ecological monitoring station has been established. In this study, only the CFERN and auxiliary observation sites in nearby areas or similar habitats were replaced, which reduces evaluation accuracy. Therefore, the next step is to establish a national ecological monitoring station for the natural forest protection project area, so that the data can be used. In the future, more and more measured data can be obtained with increases in the number of monitoring stations and determining the layout of natural forest protection project.

#### 4.2.2 Ecosystem services management

A comprehensive and systematic simulation and estimation of ecosystem services is a prerequisite for ecosystem services management and related policy formulation. The spatial heterogeneity of geographical environment will lead to uncertainty in the trade‐off and coordination of ecosystem services. This paper concludes that the trade‐off and synergy between various ecosystem services in the study area is basically equivalent, and the synergies are more significant, which also provides a good way for the region to formulate ecological restoration and protection policies. This indicates that the implementation of the NFCP has played a certain effect. In addition, there is a staggered form of trade‐offs and/or synergies between various ecosystem services, and its internal mechanism and mechanism of action need further exploration and in‐depth analysis in the future.

#### 4.2.3 Forest resource management

Forestry limited to commercial forest areas and limited cutting areas for forest annual tending is not conducive to the protection of forest resources. However, a large number of forests in the forbidden cutting area need to be tended and felled sanitarily. Without tending and sanitary felling, the growth of trees is poor, and some of the dead trees are attacked by pests and diseases. In the process of forest resources management and protection, the workers' living wages are low and the cost of forest management and protection is limited, so their ability for protecting the forest is hampered. Therefore, it is necessary to develop natural forest resources reasonably and increase forest volume. The timber output was adjusted and reduced in place according to the plan to curb the declining trend of forest resources and realize the double growth of forest resources area and stock. The government and departments of law, public security, forestry, supervision and others need to jointly and efficiently supervise and control the project in order to ensure its smooth implementation. Goals for the project include: To further improve the management and protection network of forest resources, according to the local distribution of forest resources and geographical conditions; to determine appropriate management and protection methods in line with local reality; implement the management of the project through the establishment of management stations, division of responsibility areas and other methods. In addition, a point‐to‐point cooperation mechanism between the project area and the agricultural colleges or agricultural scientific research units in the region will strengthen the prevention and treatment of natural forest diseases and insect pests.

#### 4.2.4 Capital input and management

Price indices and wage standards have risen in recent years, and the costs of forest resource management and conservation, policy expenditure and other expenses have also increased substantially. With the development of society and economy, there is a big gap between the standard of fund subsidy approved at that time and the actual expenditure on natural forest protection. The government has set up a management organization for engineering construction, and a responsibility system for engineering construction objectives has been set up from the government to the Forestry Bureau and forest farms. Project leaders should strictly control the quality of the project and the management of the use of funds, and further strengthen the assessment, project information submission and file management, to ensure that the work is in accordance with the rules and regulations.

#### 4.2.5 Staff shunt transfer

The biggest problem facing enterprises stopped after cutting is the reorienting workers (Ke et al., 2015; Yin et al., 2010). Therefore, forest industry enterprises should actively encourage employees through forest tending, management and development of the forest economy, multi‐channel development of entrepreneurial opportunities and jobs (Huang, Liu, & Tian, 2014; Zang, Zhang, & Hao, 2014). The first is to promote the sustainable management of forestry, strengthen the cultivation of reserve resources, and enhance the ability to create employment and absorb extra workers. The second is a multi‐channel placement of surplus staff and workers (Dawley, Marshall, Pike, Pollard, & Tomaney, 2014; MacKinnon, 2017), promote the reorientation of workers shunt. Third is to encourage independent entrepreneurship, entrepreneurship to promote employment, the establishment of entrepreneurship and employment "two wheeled" drive mechanism (Baumgartner, Pütz, & Seidl, 2013; 2014Parker Harris, Renko, 2014& Caldwell, 2014).

## CONFLICT OF INTEREST

None declared.

## AUTHOR CONTRIBUTIONS

Bing Wang designed the study. Xiang Niu and Peng Gao performed the analysis. Qingfeng Song helped collect the data. All authors contributed to the interpretation of the results. Longsheng Huang wrote the initial draft and refined the manuscript. Special thanks are given to the referees and the editors for their instructive comments, suggestions, and editing for the manuscript.

## Data Availability

Upon article acceptance, original data including evaluation results of ecosystem services and social public will be archived in the public repository Dryad.
